# Complete Purging of Ewing Sarcoma Metastases from Human Ovarian Cortex Tissue Fragments by Inhibiting the mTORC1 Signaling Pathway

**DOI:** 10.3390/jcm10194362

**Published:** 2021-09-24

**Authors:** Ronald Peek, Lotte L. Eijkenboom, Didi D. M. Braat, Catharina C. M. Beerendonk

**Affiliations:** Department of Obstetrics and Gynecology, Radboud University Medical Centre, 6500 HB Nijmegen, The Netherlands; Lotte.Eijkenboom@Radboudumc.nl (L.L.E.); Didi.Braat@Radboudumc.nl (D.D.M.B.); Ina.Beerendonk@Radboudumc.nl (C.C.M.B.)

**Keywords:** ovarian tissue cryopreservation, ovarian tissue transplantation, Ewing’s sarcoma, purging, Everolimus, oncofertility

## Abstract

Restoration of fertility by autologous transplantation of ovarian cortex tissue in former cancer patients may lead to the reintroduction of malignancy via the graft. Pharmacological ex vivo purging of ovarian cortex fragments prior to autotransplantation may reduce the risk of reseeding the cancer. In this study we have investigated the capacity of Everolimus (EVE), an inhibitor of the mammalian target of rapamycin complex 1 (mTORC1) signaling pathway, to eradicate Ewing’s sarcoma (ES) from ovarian tissue by a short-term ex vivo treatment. Exposure of experimentally induced ES tumor foci in ovarian tissue to EVE for 24 h completely eliminated the malignant cells without detrimental effects on follicle morphology, survival or early folliculogenesis. This indicates that effective purging of ovarian cortex tissue from contaminating ES tumor foci is possible by short-term exposure to EVE.

## 1. Introduction

Autotransplantation of cryopreserved ovarian cortex fragments has become a viable option for the restoration of fertility in women suffering from loss of reproductive potential. The technique is based on the cryopreservation of ovarian cortex tissue fragments (OTC) containing primordial follicles that are capable of restoring fertility after grafting. In contrast to oocyte or embryo cryopreservation, OTC is not dependent on ovarian activity and is therefore the only option for prepubertal girls to safeguard their reproductive potential when facing gonadotoxic treatment. The reproductive efficacy of ovarian tissue transplantation (OTT) is relatively high with approximately 26% of the autotransplantations resulting in live birth [1]. Although OTC is used to preserve fertility in patient groups expecting non-iatrogenic causes of fertility loss, such as systemic diseases requiring chemotherapy or bone marrow transplantation, the vast majority of patients that have their ovarian tissue cryopreserved consists of patients with malignant disease, expecting iatrogenic effects on their fertility potential due to their anti-cancer treatment [1]. Despite its broad applicability and high success rate, the autologous transplantation of ovarian tissue in cancer patients is not without concern. In most of these patients the ovarian tissue is harvested prior to chemotherapy leaving the potential risk of reintroducing malignancy via cancer cells present in the graft during autotransplantation. The risk of disease transmission through grafting was reported to be relatively small [1,2]. However, cancer cells, especially from hematologic malignancies, have been detected in ovarian tissue harvested for fertility preservation purposes and retain the capacity to develop metastases after xenotransplantation [1,3]. Chemotherapy administered before OTC has been reported to reduce the risk of disease transmission without significantly compromising fertility outcomes, especially in younger patients [1,4]. The development of additional, more effective procedures to prevent reintroduction of malignancy is still actively pursued [5], but so far none of these techniques have passed the experimental stage. For example, the use of mature oocytes from in vitro maturation of primordial follicles [6,7,8] or from stem cells [9,10,11,12] circumvent the risk of cancer cell reintroduction by grafting as they do not require transplantation of patient-derived tissue fragments. By separating small follicles from the potentially contaminated stroma cell compartment, followed by seeding of these purified follicles to a transplantable artificial ovary might also avoid the problem of reintroduction of malignancy [12,13,14,15]. All of these techniques are technically very challenging and take considerable time to complete. Alternatively, we and others are focusing on the ex vivo purging of ovarian cortex fragments to eradicate contaminating cancer cell prior to OTT [16,17,18]. Purging does not require fractionation of the tissue into individual cell types, is technically easy to perform and can be completed in a single day. We have shown the successful purging of ovarian tissue harboring tumor foci from rhabdomyosarcoma by short-term pharmacological inhibition of YAP/TAZ oncoproteins with Verteporfin, without compromising ovarian tissue integrity [16]. Tumor foci of chronic and acute myeloid leukemia could be eliminated from cortex fragments by ex vivo purging using a specific inhibitor of the Aurora kinases [18]. These purging experiments demonstrated that complete elimination of cancer cells from ovarian cortex fragments is possible, but also indicated that each malignancy requires a tailor-made approach with specific inhibitors. For example, purging of acute lymphocytic leukemia with dexamethasone was found to be ineffective [17], while verteporfin, a highly effective drug for the eradication of contaminating rhabdomyosarcoma cells, had no effect on breast cancer cell foci in ovarian cortex tissue [16].

In the current study we focus on the purging of ovarian cortex tissue contaminated with Ewing’s sarcoma (ES). ES is a common type of primary bone cancer that predominantly affects children and adolescents. The malignancy is characterized by a chromosomal translocation t(11;22)(q24;q12) fusing the Ewing sarcoma breakpoint region 1 (EWS) gene with the Friend leukemia integration 1 transcription factor (FLI1) gene which generates an aberrant transcription factor leading to oncogenesis [19,20]. Curative treatment consists of surgery, radiotherapy and aggressive polychemotherapy. The young age of ES patients combined with the gonadotoxic side effects of their anti-cancer therapy make that these patients frequently apply for OTC to safeguard their fertility. Similar to most solid cancers ES is not frequently found in ovarian tissue harvested for fertility preservation. In a study by Greve et al. [21] malignant cells were not detected in ovarian tissue stored for fertility preservation of nine surviving ES patients. Despite that metastatic involvement of the ovaries is not common, infiltration of ovarian tissue with ES has been reported by several authors [22,23,24,25,26], also in the context of OTC [27,28].

The aim of the current study was to develop an ex vivo purging protocol specifically for the eradication of contaminating ES cells from ovarian cortex fragments to prevent reseeding of the cancer. To this end we expanded a previously described tumor model system to mimic the correct physiological in vivo microenvironment of ES metastases in human ovarian cortex tissue [16,18,29]. For pharmacological intervention we used Everolimus (EVE), a potent protein kinase inhibitor of the mammalian target of rapamycin complex 1 (mTORC1) serine/threonine kinase signal transduction pathway. mTORC1 is associated with uncontrolled cell proliferation, increased cell survival and aberrant angiogenesis [26]. The expression of mTORC1 is frequently deregulated in cancer, including ES and is therefore a promising target for purging ovarian tissue from contaminating ES metastases [26,30,31]. A significant additional benefit of mTORC1 inhibition is that it prevents unwanted culture-induced primordial follicle activation and subsequent follicular burnout that might occur during the short-term culture intrinsic to the ex vivo purging protocol [32,33,34,35]. We demonstrate that a 24 h ex vivo exposure to EVE completely eliminates Ewing’s sarcoma metastases harboring different EWS-FLI1 translocations from ovarian cortex fragments without affecting the ovarian follicles and stromal cells.

## 2. Materials and Methods

### 2.1. Human Ovarian Tissue

Intact human ovaries were obtained after written informed consent by laparoscopic oophorectomy from 14 transgender persons (aged 18–26) during gender affirming surgery. Directly after oophorectomy the ovaries were transported on ice in L15 medium (BioWhittaker Lonza, Basel, Switzerland) to the laboratory. Ovarian cortex fragments measuring approximately 6 mm × 4 mm × 1 mm were prepared the same day and cryopreserved following clinical standards using a slow-freezing protocol and dimethyl sulfoxide (DMSO; WAK- Chemie Medical GmbH, Steinbach, Germany) as a cryoprotectant. Directly before use, ovarian fragments were thawed and washed three times in culture medium to remove the cryoprotectant [36].

### 2.2. Ewing’s Sarcoma Cell Lines

Patient-derived Ewing’s sarcoma cell lines ES2, ES7 [37], CHP-100 [38] and EW8 [39], all express functional transcripts from the EWS-FLI1 fusion gene and have been widely used for Ewing’s sarcoma related research. Cell lines were cultured in Dulbecco’s Modified Eagle’s medium (DMEM, BioWhittaker, Basel, Switzerland) supplemented with 10% Fetal Bovine Serum (FBS, Gibco, Life Technologies, Carlsbad, CA, USA) and 40 µg/mL gentamicin (Centrafarm, Breda, The Netherlands).

### 2.3. Tumor Induction

The development of a tumor-induction model based on micro-injection of cancer cells in human ovarian cortex fragments has been described in detail [29]. The model has been successfully used to establish tumor foci of rhabdomyosarcoma, breast cancer, and myeloid leukemia [16,18]. Compared to other malignancies Ewing’s sarcoma cells were able to form small metastases relatively fast in ovarian cortex tissue. In order to mimic the in vivo situation more closely tumor formation was therefore limited to only 4 days before purging.

### 2.4. Ex Vivo Purging of Ovarian Tissue Containing Ewing’s Sarcoma Metastases with Everolimus

After formation of ES metastases, the cortex fragments of six individuals were cut in two equal parts. One part was purged by exposing the tissue for 24 h to 50 µM Everolimus (Selleckchem, Pittsburgh, PA, USA) dissolved in DMSO, the other part to the same concentration of solvent in 5 mL of culture medium. After purging the tissue was transferred to a fresh plate and washed four times, 10 min each, at 37 °C in 5 mL of fresh DMEM with continuous shaking for optimal diffusion to remove the inhibitor. Next, the fragments were cultured in medium for an additional 6 days to allow any remaining ES cells to form new tumor foci.

### 2.5. Histology

Ovarian cortex fragments were first fixed for 1 h in Bouin’s solution (Klinipath, Duiven, The Netherlands), washed with water and subsequently fixed in 4% formaldehyde solution (Klinipath, Duiven, The Netherlands). After embedding in paraffin, 4 µm of the Everolimus treated tissues were completely sectioned and every sixth section was stained by standard hematoxylin eosin staining (Tissue Tek^®^ Prisma™, Sakura, Alphen aan de Rijn, The Netherlands), examined by light microscopy and photographed.

### 2.6. Immunohistochemistry

Foci of ES cells in ovarian tissue were visualized by staining for CD99 (clone O13; Thermoscientific, Waltham, MA, USA). Cell proliferation was analyzed by staining for the proliferation marker Ki-67 (clone MIB-1, Dako Agilent, Santa Clara, CA, USA). Cells in apoptosis were detected by antibodies against active Caspase-3 (clone C92-605; BD Biosciences, Franklin Lakes, NJ, USA). Antigen retrieval, incubation with primary and secondary antibodies and visualization of target–antibody interaction has already been described in detail [16,18].

### 2.7. RNA Isolation and RT-PCR

After purging the ovarian tissue was manually cut into small, approximately 1 mm^3^ fragments using a scalpel. Total RNA was isolated from these fragments using the RNA Bee kit (Tel-Test Inc., Friendswood, TX, USA). RNA was quantified using the NanoDrop 2000 spectrophotometer (Thermo Scientific, Breda, The Netherlands). First strand cDNA was synthesized by Superscript II (Invitrogen, Waltham, MA, USA) from 1 µg of total RNA using random hexamer primers (Promega, Madison, WI, USA) in 40 µL. PCR reactions were performed in 25 µL containing 2 µL of cDNA and 5 pmol of each forward and reverse primer. Primer sequences for the amplification of the EWS-FLI1 fusion transcript were 5′TCCTACAGCCAAGCTCCAAGTC3′ and 5′CTGGAGAGCGAGGTGGCTTC3′ (forward primers in exons 7 and 9 of the EWS gene) and 5′CTGATCGTTTGTGCCCCTCC3′ (reverse primer in exon 7 of the FLI1 gene). PCR was run in a T100 thermocycler (Biorad, Lunteren, The Netherlands) for 10 min at 95 °C, followed by 35 cycles of 92 °C for 1 min (denaturation), 65 °C for 1 min (annealing) and 2 min at 72 °C (extension). The HBMS control transcript was RT-PCR amplified with primers 5′TGCCAGAGAAGAGTGTGGTG3′ and 5′ATGATGGCACTGAACTCCTG3′ using the same program but with an annealing temperature of 60 °C. PCR products were run on a standard 1.5% agarose gel and photographed.

### 2.8. Assessing the Viability of Ovarian Tissue and Follicles after Purging

The viability of the tissue after purging was analyzed by three different assays: standard Hematoxylin Eosin (HE) staining to determine the percentage of morphologically damaged follicles, Neutral Red (NR) staining to assess the percentage of viable small follicles and by an In Vitro Growth assay (IVG) to analyze the capacity of small follicles to progress to secondary follicles. Morphology of HE stained follicles was examined as described [16,40]. Staining of follicles with NR has been published in detail elsewhere [18,41]. In brief, follicles from cortex tissue exposed to EVE and control tissue were released from the surrounding tissue by collagenase treatment followed by staining with Neutral Red (Sigma-Aldrich, Saint Louis, MO, USA) and examined by light microscopy. Viable follicles stain red while nonviable follicles remain colorless. The capacity of small follicles to initiate folliculogenesis after purging was assessed by applying the first step of an in vitro maturation protocol described by McLaughlin et al. [6]. The treated tissue was cut into 1 mm × 1 mm × 0.5 mm fragments and cultured for 8 days in IVG medium to allow small follicles to develop to secondary follicles. Subsequent steps of this protocol require manual dissection of the secondary follicles from the stromal tissue and are not suitable for quantitative analysis. After IVG, the tissue fragments were HE stained and the follicles were categorized according to their maturation stage as described [42,43].

## 3. Results

### 3.1. Establishment of an Ex Vivo Tumor Model for Ewing’s Sarcoma Metastases in Human Ovarian Cortex Fragments

**Figure 1:**
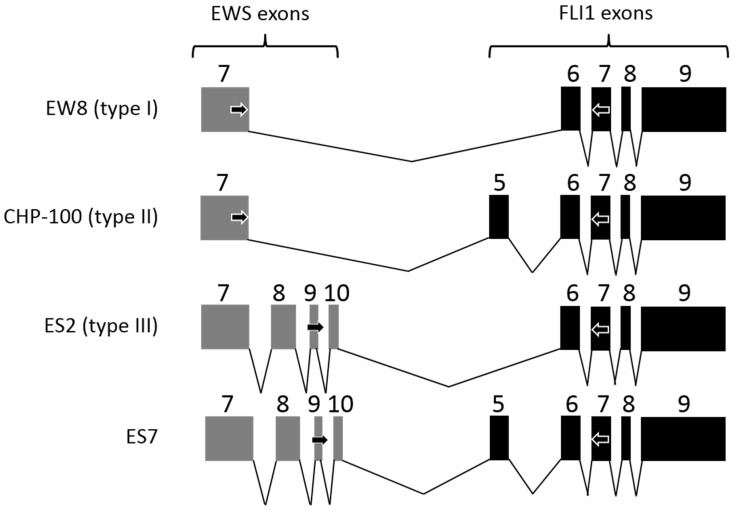
The t(11;22)(q24;q12) chromosome translocation in the ES cell lines. Schematic representation of the t(11;22)(q24;q12) reciprocal chromosomal translocation involving the EWS and FLI1 gene in the four cell lines (EW8, CHP-100, ES2 and ES7) that were used to induce metastatic foci of Ewing’s sarcoma in human ovarian cortex tissue fragments. EW8 contains the most common fusion between the EWS gene exon 7 with FLI1 gene exon 6 (type I). The location of the reverse PCR primer in exon 7 of the FLI1 gene and the two forward primers located in exons 7 and 9 of the EWS gene for the detection of the EWS-FLI1 fusion transcripts are indicated by arrows. CHP, Children’s Hospital of Philadelphia; ES, Ewing’s sarcoma; EWS, Ewing sarcoma breakpoint region 1; FLI1, Friend leukemia integration 1 transcription factor.

To obtain sufficient tissue containing small ES metastases for our purging experiments we aimed to establish an ex vivo tumor model as previously developed for several other malignancies [16,18,29]. We selected four patient-derived ES cell lines for introduction into ovarian cortex fragments. These cell lines cover the most common types of translocations in ES between the EWS and the FLI1 gene (Figure 1) [44].

**Figure 2:**
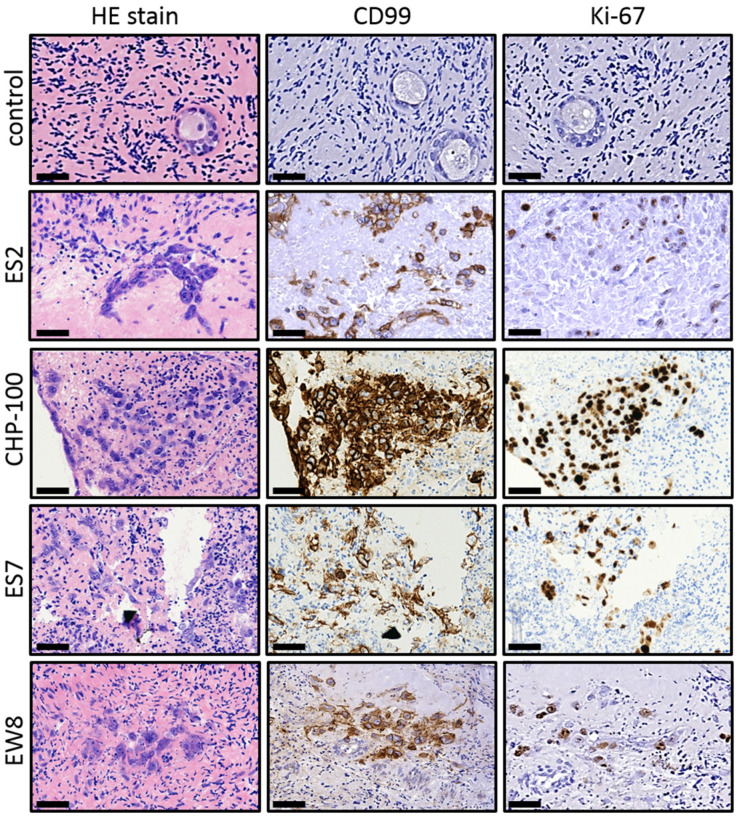
Establishing an organ culture system harboring tumor foci of Ewing’s sarcoma. After introduction into human ovarian cortex tissue followed by 4 days of culture, cells of ES2, CHP-100, ES7 and EW8 formed small tumor foci that could be easily distinguished from the surrounding ovarian tissue by standard HE staining. Staining for the membrane protein CD99, a marker for ES that is not expressed in ovarian cortex tissue (control), revealed that malignant cells were capable of establishing new tumor foci at distant sites during culture. Part of the ES cells were positive for the Ki-67 proliferation marker, indicating that tumor foci were still growing at the end of the culture period. Bars indicate 25 µm. EW, Ewing; HE, Hematoxylin Eosin; CD, cluster of differentiation.

After introduction in the tissue the ES cells were allowed to form tumor foci for 4 days before the tissue was evaluated by (immuno) histochemistry (Figure 2). All four cell lines were capable of establishing foci and ES cells could be readily distinguished from the surrounding ovarian tissue by their size and HE staining intensity. The presence of ES cells was confirmed by staining of CD99, a membrane protein expressed by ES cells but not present in the control tissue. Part of the ES cells in the tumor foci expressed Ki-67, indicating ongoing tumor cell proliferation. In addition, we observed numerous very small foci and single ES cells adjacent to larger more densely packed tumor foci (mainly in ES2 and ES7), indicating that ES cells were capable of migrating in the surrounding tissue and establishing new foci. These results indicate that the introduction of patient-derived ES cells into human ovarian cortex results in the formation of mitotically active tumor foci of different sizes that are in close contact with the ovarian tissue.

### 3.2. Purging Ovarian Cortex Fragments of Ewing’s Sarcoma Metastases by Treatment with Everolimus

**Figure 3:**
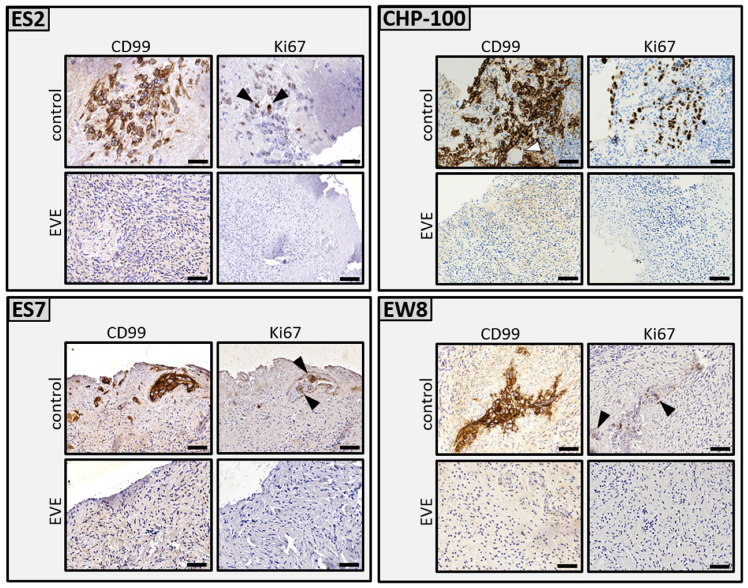
Ex vivo purging of ovarian cortex tissue from tumor foci of Ewing’s sarcoma. Ovarian cortex fragments from five individuals containing proliferating tumor foci of ES cell lines ES2, CHP-100, ES7 and EW8 were cut into two equal parts. One part was treated with solvent-only (control), the other part with 50 µM EVE. After purging and washing the tissue fragments were cultured for an additional 6 days to allow any remaining ES cells to form new tumor foci. Immunohistochemical staining of serial histological sections throughout the tissues revealed CD99 and Ki-67 positive tumor foci in the control tissue but not in the EVE treated tissue. Although some sections of the EVE treated tissue contained ES cell remnants, no CD99 positive tumor cells with normal morphology were detected. Arrowheads point to Ki-67 positive cells in the control tissue. Bars indicate 25 µm. EVE, Everolimus.

We first tested the effect of EVE on ES cells grown on tissue culture plastic. Although a 24 h treatment with 5 µM of EVE had effect on morphology and plastic adherence of the cells, a concentration of 50 µM was required to fully eradicate the ES cells (results not shown). In all subsequent experiments a 24 h incubation of EVE at a concentration of 50 µM was used. Next, we investigated the tumoricidal capacity of EVE in our ex vivo tumor model of ES in ovarian cortex tissue fragments. EVE treatment of the tissue, followed by an additional 6 days of culture to allow any remaining ES cells to form new foci, showed a complete eradication of ES cells, as evidenced by the absence of CD99 and Ki-67 positive cells. The solvent-treated control tissue showed numerous CD99/Ki-67 positive tumor foci (Figure 3).

**Figure 4:**
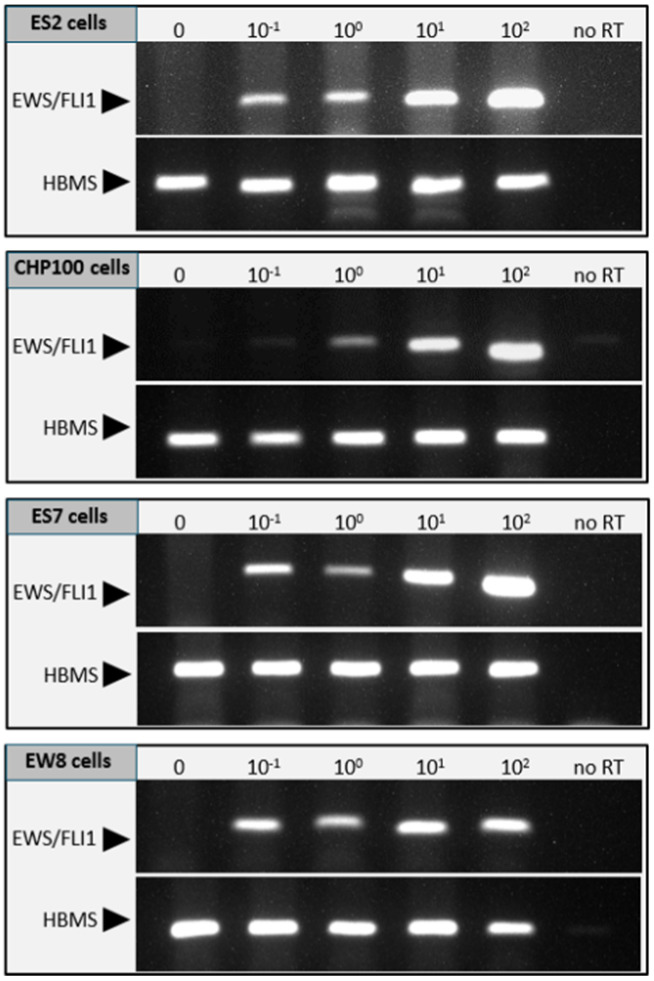
Determining the sensitivity of the RT-PCR for the detection of the tumor specific EWS-FLI1 fusion transcript. The lower limit of detecting ES tumor cells in the context of ovarian cortex tissue was determined by spiking tissue fragments with a set number of cells (0, 0.1, 1, 10 or 100 cells) from cell lines ES2, CHP-100, ES7 or EW8. After RNA isolation and reverse transcription, the first strand cDNA was used for PCR to detect the EWS-FLI1 fusion transcript. Agarose gel electrophoresis of PCR products showed that the lower limit of detection for CHP-100 was 1 cell, for ES2, ES7 and EW8 it was less than 1 cell. In RNA from ovarian tissue without ES cells or with spiked EWS cells but without a reverse transcription reaction (no RT), the EWS-FLI1 fusion transcript was not detected by PCR. Expression of the housekeeping gene hydroxymethylbilane (HBMS) was used to normalize EWS-FLI1 signals.

The sensitivity of (immuno)histochemistry is limited for the detection of any remaining small tumor foci or individual malignant cells in the tissue after purging. We therefore employed an RT-PCR for the detection of the ES specific EWS-FLI1 gene fusion transcripts to further confirm the absence of ES cells after treatment with EVE. The sensitivity of the EWS-FLI1 specific RT-PCR was determined by spiking ovarian cortex fragments with a set number of ES cells and was found to be less than 1 cell for the cell lines ES-2, ES7 and EW8 and 1 cell for CHP100 (Figure 4).

**Figure 5:**
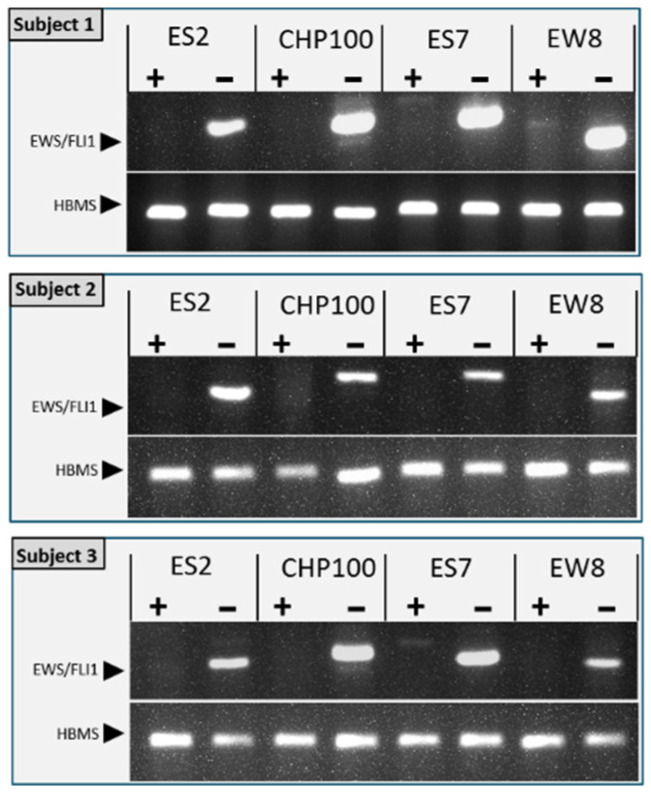
Detection of the ES specific EWS-FLI1 fusion transcript in purged ovarian cortex tissue. Ovarian cortex tissue fragments from three individuals with tumor foci of cell lines ES2, CHP-100, ES7 or EW8 was cut into two equal parts. One part was treated for 24 h with 50 µM EVE, the other part with solvent-only. After washing and an additional culture for 6 days total RNA was extracted from the tissue fragments and assessed for the presence of the ES specific EWS-FLI1 fusion transcript by RT-PCR. The fusion transcript was detected in all tissues treated with solvent-only, but not after treatment with EVE. In some lanes (EW8+ and ES7+), faint bands are visible, but these are at different positions in the gel than the EWS-FLI PCR products. Expression of the housekeeping gene hydroxymethylbilane (HBMS) was used as a control.

Subsequently, we used this RT-PCR to assess the purging efficiency of ovarian tissue containing ES tumor foci. The absence of the EWS-FLI1 translocation transcript after purging confirmed the complete eradication of ES cells from the tissue after EVE treatment (Figure 5).

### 3.3. Purging with Everolimus Does Not Impair Ovarian Tissue Integrity

**Figure 6:**
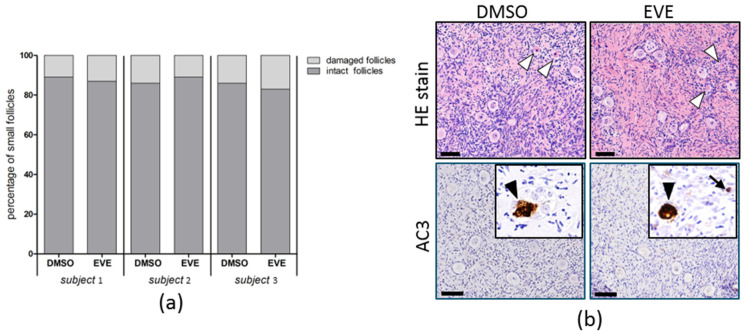
Follicular morphology after 24 h of purging with Everolimus. (**a**) To determine the possible adverse effects of EVE on follicles, ovarian cortex tissue of three individuals was exposed for 24 h to 50 µM EVE or solvent only. After washing the tissue was cultured for an additional 24 h to allow any morphological defects to become apparent. Per condition at least 100 follicles were analyzed. The percentage of morphologically intact small follicles of tissue after EVE exposure was very similar to the control tissue. (**b**) Representative images of HE staining of tissue treated with EVE or solvent-only showing many small follicles with normal morphology and some follicles with damaged pyknotic/eosinophilic oocytes (white arrowheads). Immunostaining of active Caspase-3 (AC3) was used to determine the presence of apoptotic ovarian cells after purging. Apoptotic cells were virtually absent (insets) with only very few positive oocytes (black arrowheads) or stromal cells (black arrow). Bars indicate 100 µm. DMSO, dimethyl sulfoxide.

To investigate the possible detrimental effects on the ovarian tissue of a 24 h exposure to EVE we determined the number of morphologically intact small (primordial and primary) follicles, the number of viable follicles, the presence of apoptotic cells and the capacity of small follicles to progress to secondary follicles. The percentage of morphologically damaged small follicles was not affected by purging with EVE and similar to the control tissue (Figure 6a). Staining for active Caspase-3 indicated that apoptotic cells were almost absent and positive stromal or follicular cells were found only sporadically in both treated and control tissue (Figure 6b).

**Figure 7:**
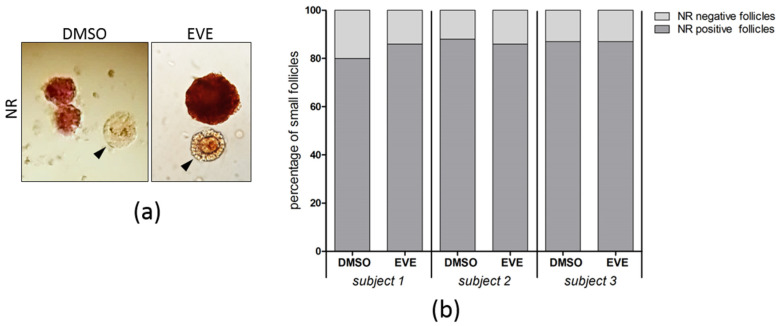
Follicular viability after 24 h of purging with Everolimus. (**a**) Ovarian tissue from 3 individuals exposed for 24 h to EVE or solvent only was cultured for an additional 24 h and assayed by Neutral Red (NR)staining of follicles. For each condition 100 small follicles were evaluated. Follicles staining bright red were considered intact and viable, unstained or very faint stained follicles (black arrowheads) were scored as damaged. (**b**) The percentage of Neutral Red positive and negative small follicles was comparable for tissue purged with EVE or tissue treated with solvent only.

Viability of follicles was analyzed by a Neutral Red staining assay and showed no significant differences between tissue exposed to EVE and control tissue (Figure 7).

**Figure 8:**
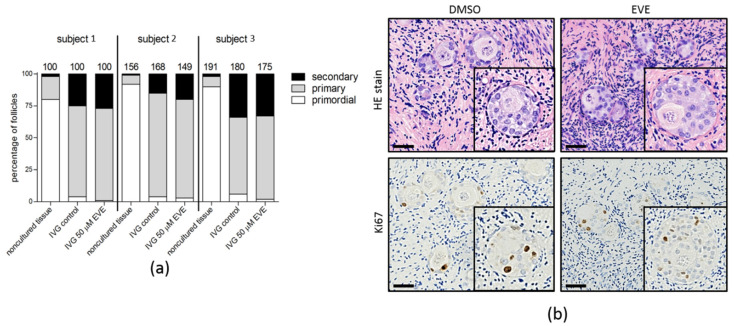
Capacity of small follicles to start folliculogenesis after purging with Everolimus. (**a**) Ovarian cortex tissue from three individuals was exposed for 24 h to EVE or solvent only. After removing the inhibitor, the tissue was cultured in in vitro growth (IVG) medium for 8 days. After IVG the percentage of primordial, primary and secondary follicles was determined in at least 100 follicles from non-cultured tissue, tissue purged with EVE and tissue treated with solvent-only (top panel). During IVG an increase in the percentage of primary and secondary follicles was observed compared to the non-cultured tissue in all three subjects, albeit with different efficacies. The percentage of the different stages of follicular development was very similar between the EVE treated tissue and the control tissue after IVG. The number of follicles analyzed is indicated above the bars. (**b**) HE staining of tissue sections after IVG showed secondary follicles with a multilayer of granulosa cells up to 150 µm in diameter. Immunostaining for the proliferation marker Ki-67 showed that granulosa cells of secondary follicles were still mitotically active at day 8 of IVG. Bars represent 40 µm.

The capacity of small follicles to progress to secondary follicles was analyzed by an In Vitro Growth assay (IVG; Figure 8). In non-cultured tissue the percentage of small follicles varied between 80 and 92%, with only very few secondary follicles. After 8 days of IVG a clear shift from primordial to primary and secondary follicles was observed. The number of primordial follicles was reduced to 1–6% while numbers of primary and secondary follicles increased to 60–81% and 20–34% of the total number of follicles, respectively. At day 8 of IVG folliculogenesis was still ongoing as evidenced by the presence of Ki-67 positive granulosa cells in secondary follicles of both tissue exposed to EVE and control tissue. Although the percentages of the different follicle stages varied between individuals, no significant differences were observed between the EVE exposed tissue and the control tissue.

Taken together, these results indicate that purging of ovarian cortex tissue from malignant cells by a 24 h ex vivo treatment with EVE does not impair ovarian cortex tissue integrity.

## 4. Discussion

In this paper we provide evidence that ovarian cortex tissue harvested for fertility restoration purposes can be purged completely from Ewing’s sarcoma metastases by an ex vivo pharmacological inhibition of mTORC1 with EVE. Ovarian tissue integrity, and more specifically ovarian primordial follicle morphology, viability and capacity to progress to secondary follicles was not affected by purging.

The ovary is not a common site for ES to metastasize [45]. However, ovarian involvement of ES or ES related malignancies has been described in several case reports [22,23,24,25,46,47]. A study by Greve et al. [21] provided no evidence for ovarian involvement in tissue stored for fertility preservation from nine patients with ES. Abir et al. [27] analyzed the ovarian tissue from eight Ewing’s sarcoma patients and found evidence of ovarian involvement in one patient by RT-PCR amplification of the ES specific translocation transcript. Infiltration of ovarian tissue cryopreserved for fertility preservation purposes with ES was also described in two young patients, based on the presence of CD99 positive tumor cells harboring the EWS-FLI1 translocation [28]. Although these studies suggest that ovarian involvement in ES is relatively rare, the frequency at which ovarian tissue fragments are contaminated with ES cells might be underestimated. Usually only one or a few tissue fragments are routinely analyzed for the presence of malignant cells before grafting, while the rest of the fragments that are actually transplanted are not examined. Cortex tissue fragments from the same ovary may give different results when analyzed for contamination with malignant cells [48] and indicate that negative results for only a small part of the cortex may not be representative for the remaining tissue fragments.

The development of an effective and safe purging protocol for the ex vivo eradication of ES requires a large number of human ovarian cortex fragments containing ES metastases. Clearly, these are not available in sufficient numbers from ES patients that have cryopreserved their ovarian tissue for fertility preservation. Furthermore, ovarian involvement in ES patient derived tissue can only be established by first examining the tissue by histology or molecular techniques that will render the tissue unsuitable for purging experiments. We therefore successfully expanded our previously established tumor induction model based on the micro-injection of malignant cells into fragments of human ovarian cortex to obtain tissue harboring small ES metastases [16,18,29]. This provided us with sufficient tissue fragments containing proliferating, metastasis-like ES tumor foci for our purging experiments. Remarkably, ES cells not only gave rise to tumor cell foci at the site of injection but appeared to migrate in the surrounding tissue and established new tumor foci at distant sites during the culture period, mimicking the in vivo situation even more closely. As a source of human ovarian cortex tissue, we used ovarian tissue recovered from transmen after bilateral ovariectomy. These individuals are generally young and their ovarian tissue is an excellent source for research because of normal morphology and the presence of large numbers of primordial follicles [49,50]. The patient-derived ES cancer cell lines used to establish tumor foci in the ovarian tissue all contained an EWS-FLI1 translocation, including the most common functional gene fusion of EWS exon 7 with FLI1 exon 6 (type I) [44] in cell line EW8. The use of cell lines allowed us to perform the required culture experiments that would have been very difficult, if not impossible, to achieve with primary cancer cells. ES cancer cell lines have been used extensively for research purposes including the development of new drugs [51], characterization of drug resistance in ES [52], the effects of pharmacological treatment [39] and even for the development of clinical treatment regimens (EWING2008; ClinicalTrials.gov Identifier: NCT00987636). To study the purging effect of EVE it is essential that the ES metastases are in the appropriate microenvironment. Susceptibility of cancer cells to tumoricidal agents is highly dependent on the interaction between cancer cells and extra-cellular matrix (ECM) of the surrounding tissue [53]. The ovarian cortex has a dense and rigid ECM which may shield the ES metastases from EVE and may constitute a barrier for the diffusion of oxygen and nutrients leading to the activation of drug resistance pathways [53]. Previous purging experiments assessing the use of Verteporfin to eradicate malignant cells from ovarian cortex also gave evidence of the importance of performing purging experiments in the correct microenvironment [16]. Expression of mTORC1, the target of EVE treatment, is dependent on mechanical stretch, again stressing the role of tissue ECM on cancer cell susceptibility to EVE [54]. These observations justify the use of our organ culture system which provides a tumor microenvironment that is identical to the in vivo situation, with tumor cells that are in close contact with the ovarian ECM. We therefore consider our organ culture system a valuable model to study the effect of pharmacological ex vivo purging of human ovarian cortex tissue from ES metastases. Despite the many similarities between our organ culture system and the in vivo situation our results should be interpreted with caution since no ovarian tissue derived from ES patients with metastatic disease was used.

The effectiveness of purging ovarian cortex tissue from contaminating ES metastases with EVE was determined by serial sectioning of treated tissue followed by (immuno) histochemical staining of a sections with 20 µm intervals. Although this method has been described to detect a single tumor cell metastasis [55], sensitivity of histology for the detection of very small tumor foci or single cancer cells is low. To ensure that no living ES cells were present in the tissue after purging we used an EWS-FLI1 transcript specific RT-PCR, capable of detecting a single ES cell in the context of ovarian cortex tissue. This molecular analysis confirmed the results obtained with histology and showed that a 24 h ex vivo exposure to 50 µM of EVE is a highly effective treatment for the eradication of ES metastases from ovarian cortex fragments.

In addition to effective elimination of ES metastases from ovarian cortex tissue the purging treatment should not compromise the functional integrity of the ovarian cortex tissue. To verify that the ovarian follicles and stromal cells were not affected by the short-term exposure to EVE we performed several assays to monitor follicular morphology, follicular viability, apoptosis and the capacity of small follicles to progress to secondary follicles. None of these assays showed any evidence of harmful effects. This indicates that ex vivo purging for 24 h with EVE may not impair ovarian cortex tissue function.

The purging protocol requires a 24 h culture period of the ovarian tissue during the exposure to EVE. Although short term culture of ovarian tissue fragments prior to OTT has been performed before in a clinical setting and resulted in live birth [56], culturing of ovarian tissue may lead to massive and premature activation of resting follicles resulting in a considerable depletion of the follicular pool. Interestingly, a 24 h culture of ovarian cortex tissue in the presence of EVE has been described to prevent the spontaneous activation of small follicles in both rodent and human tissue [32,34,35,57,58,59]. This indicates that EVE is capable of not only effectively eliminating ES metastases, but also protects the tissue from so-called follicular-burnout during culture. In our in vitro growth experiments, the inhibiting effect of EVE on folliculogenesis was no longer present after extensive washing of the tissue fragments because small follicles were capable of resuming folliculogenesis and progressed to secondary follicles. This is consistent with the observations that mTOR pathways are not involved in the viability of primordial follicles but are essential in more advanced stages of folliculogenesis [60]. The concentration of 50 µM EVE in the culture medium we used for the eradication of ES metastases was considerably higher than for the protection against follicular burnout, which was already reached at the nanomolar range. However, in a mouse model of acute lymphoblastic leukemia EVE concentrations were also in the micromolar range for clearance of the disease [61], while effective growth inhibition of primary breast cancer cells required exposure to at least 10 µM of EVE [62]. This suggests that relatively high concentrations of EVE are needed for effective killing of cancer cells compared to the concentration required for the prevention of follicular-burnout during culture.

EVE has been FDA approved for first- and second line cancer treatment including renal cell carcinoma, breast cancer, astrocytoma and several other malignancies [25]. In metastatic Ewing sarcoma EVE is used as a second line option [63]. Systemic use of EVE may have serious adverse side effects, including lesions within the oral mucosa, stomatitis and gastrointestinal distress [64]. These side effects are consistent with the indispensable role of mTOR1 signaling in homeostasis of healthy tissue. Obviously, these adverse effects of systemic EVE treatment do not occur in our organ culture system.

## 5. Conclusions

This study provides evidence that short term ex vivo exposure of ovarian cortex tissue contaminated with ES metastases to EVE results in complete elimination of malignant cells without impairing tissue integrity. In addition, EVE may exert a transient protective effect on the tissue during purging by preventing culture-induced primordial follicle activation.
